# Targeting Cytokine Storm in COVID-19: A Role of Online Hemodiafiltration with Asymmetric Cellulose Triacetate in Maintenance Hemodialysis Patients—A Report of 10 Cases

**DOI:** 10.1155/2021/5575928

**Published:** 2021-03-12

**Authors:** José C. De La Flor, Francisco Valga, Alexander Marschall, Tania Monzon, Cristina Albarracín, Elisa Ruiz, Miguel Rodeles

**Affiliations:** ^1^Department of Nephrology, Central Defense Gomez Ulla Hospital, Madrid, Spain; ^2^Department of Nephrology, Doctor Negrín University Hospital, Las Palmas de Gran Canaria, Spain; ^3^Department of Cardiology, Central Defense Gomez Ulla Hospital, Madrid, Spain; ^4^Department of Hemodialysis, Avericum S.L., Las Palmas de Gran Canaria, Spain

## Abstract

Early reports have suggested that maintenance hemodialysis (MHD) patients could be more susceptible to a severe course of COVID-19. Among the therapeutic approaches, the use of drugs that reduce the cytokine storm characteristic of this disease has been proposed. Some dialyzers, such as the new generation of asymmetric cellulose triacetate (ATA) membranes, could favor the effective elimination of medium-sized molecules and other inflammatory mediators. In this case series, we describe in depth the clinical, analytical, and radiological details, therapeutic aspects, and outcomes of the case series of 10 MHD patients of our dialysis unit, who tested positive for SARS-CoV-2 from 5 October to 30 November 2020. Furthermore, we evaluate the removal of hyperinflammatory parameters with the ATA membrane in postdilution online hemodiafiltration (OL-HDF) in these patients through a variety of biomarkers of systemic inflammation from the diagnosis until stripping. Biochemical blood analysis was carried out at baseline and at days 7 and 14 after diagnosis, respectively. 50% of the patients presented COVID-19 pneumonia and required hospital admission. Median hospitalization time was 21 days. A total of 4 patients developed severe pneumonia (3 of them died) and 1 patient developed moderate pneumonia. Patients who died (*n* = 3) were more likely to present bilateral pneumonia (100% vs 14.3%) at diagnosis and less reduction in interleukin 6 (IL-6) at day 14, as compared to those who survived. The use of the ATA membrane could be considered a therapeutic option, due to its immunomodulatory effect in MHD patients with SARS-CoV-2 infection, especially at the beginning of the disease, where the inflammatory component is predominant.

## 1. Introduction

In early December 2019, a respiratory disease caused by a novel coronavirus, named severe acute respiratory syndrome coronavirus 2 (SARS-CoV-2), emerged in Wuhan, China. This disease, which the World Health Organization (WHO) denominated coronavirus disease 2019 (COVID-19), spread rapidly throughout China and worldwide [1]. The WHO declared COVID-19 a pandemic in March 2020 [2]. At the beginning of June 2020, Spain was already recovering from the high infection rates, after it implemented one of the most draconian COVID-19 lockdowns in Europe. However, two months after lifting the restrictions, the virus spread faster than in any of Spain's neighboring countries. During the second wave of COVID-19, the therapeutic approach of targeting the virus replication and the hyperinflammation remains limited and its pathophysiologic basis is not yet not fully understood. Patients with end-stage kidney disease (ESKD), undergoing maintenance hemodialysis (MHD), may be more vulnerable to a SARS-CoV-2 infection, due to their suppressed immune system [3, 4]. Prompt identification of SARS-CoV-2 infection, isolation, and treatment according to the different manifestations of the disease are essential to reduce the spread of the disease. This is especially important in this population, given the logistical aspects of MHD that may increase the risk for disease transmission [5].

Among the therapeutic options for COVID-19, the use of drugs that reduce the hyperinflammation (cytokine storm) has been proposed [6–9]. The ERA/EDTA recommends in patients undergoing hemodialysis the use of the medium cut-off membranes (MCO) filter, with the aim of increasing the efficiency of removal of middle size molecules and, therefore, inflammation mediators implicated in the pathogenesis of SARS-CoV-2 infection, as established by the “Brescia Renal COVID-19 Task Force” [10]. However, their efficacy and safety are not been validated so far. After the first outbreak of SARS-CoV-2 in our dialysis unit [11], we established an action protocol for the management of patients in MHD with COVID-19. We used postdilution online hemodiafiltration (OL-HDF) with a dialyzer of asymmetric cellulose triacetate (ATA) membrane. This is a new generation of the cellulose triacetate membrane, with an increase in the coefficient of ultrafiltration (72 mL/h/mmHg) [12]; this could favor the elimination of medium-sized molecules and other inflammatory mediators. The aim of the present study was to describe in depth the clinical, analytical, and radiological details, therapeutic aspects, and outcomes of the case series of patients diagnosed with COVID-19 in our dialysis unit. In addition, we evaluate the removal of hyperinflammatory parameters with the ATA membrane in MHD patients with COVID-19, through a variety of biomarkers of systemic inflammation (neutrophil-to-lymphocyte (NLR), platelet-to-lymphocyte (PLR), monocyte-lymphocyte (MLR) lymphocyte-to-C-reactive protein (LCR) ratios, and interleukin 6 (IL-6)).

## 2. Case Reports

This is a case series of 10 adult MHD patients (13.2%) that tested positive for SARS-Cov-2 infection in the dialysis unit (with a total of 76 patients) of the Central Defense Gomez Ulla Hospital in Madrid, Spain. The patients were diagnosed from 5 October to 30 November 2020. The diagnosis was established according to the Spanish health system diagnostic criteria, which includes a positive result of the nasopharyngeal swab for SARS-CoV-2 nucleic acid, using reverse transcription-polymerase chain reaction (RT-PCR) [13]. If the RT-PCR resulted negative, however, the clinical suspicion remained strong, a second RT-PCR and/or a thoracic CT scan were performed. RT-PCR assay was performed according to the manufacture´s protocol by our center´s laboratory using Seegene's COVID-19 test kit. All SARS-Cov-2-infected patients received 3 HD sessions a week with the ATA membrane in postdilution OL-HDF and venous line administration of low-molecular-weight heparin (LMWH, Enoxaparin) at a dose of 40 to 60 mg at every HD session in nonhospitalized patients and a dose of 1 mg/kg/day (maximum dose of 80 mg) in hospitalized patients until stripping.

Initially, suspected or confirmed SARS-CoV-2 infected patients were dialyzed in a single isolation room. The patients were deisolated after two negative nasopharyngeal PCR tests taken 48 hours apart, obtained at days 14 and 15, or only one RT-PCT test if negative IgM and positive IgG results from the SARS-CoV-2 serological test. We collected demographic and epidemiological information, clinical features at diagnosis, and chest X-rays as well as technical parameters of dialysis and vascular access (Table 1). The treatments and outcomes are described in Table 2. For each patient, a blood test for systemic inflammation biomarkers was performed every 7 days from the diagnosis of infection Day 0 to Day 14. IL-6 analysis was performed at the time of diagnosis and 15 days after the start of the first session with the ATA membrane in OL-HDF.

The median age was 72 years (IQR: 69–80), and 50% were women. Fever and cough resulted to be the most prevalent symptoms (Table 1). According to the classification of the consensus document of the Spanish health system, half of the patients had COVID-19 pneumonia and required hospital admission. Four patients developed severe pneumonia (3 of them died) and 1 had moderate pneumonia. The median hospitalization time was 21 days. The 3 patients who died required a prolonged in-hospital stay of 24 days due to bacterial superinfection. Patients who died presented more often with fever than survivors (100% versus 43%, *p* value = 0.200), cough (100% vs 57.1%, *p* value = 0.475), diarrhea (66.7% vs 0%, *p* value = 0.067), and bilateral pneumonia (100% vs 14.3%, *p* value = 0.040) at diagnosis (Table 1).

**Figure 1:**
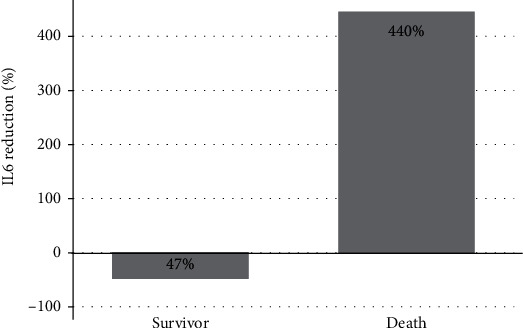
Interleukin-6 level reduction at 14 days according to vital status.

Among the analytical parameters, we found no significant differences with regard to the laboratory data at 14 days and compared to baseline except for the white blood cell (WBC) count and NLR. See Table 3 for details. At diagnosis, the patients who died had higher mean C-reactive protein (CRP) levels (1.7 versus 0.8 mg/L) and higher IL-6 levels (41.4 versus 31.1 pg/mL), as compared to survivors, however, not reaching statistical significance. The median LCR at 7 (0.5 vs 0.9, *p* value = 0.017) and 14 days (0.4 vs 3.04, *p* value = 0.033) was lower in patients who died as compared to those who survived. We also found a higher reduction in IL-6 at 14 days in patients who survived (47% vs increases of 440%) as compared to those who died (Figure 1). The rest of the analytical parameters are presented in Table 4.

With regard to the treatment of the SARS-Cov-2 infection, high flow oxygen therapy >30 l.p.m. was prescribed in 4 patients, and dexamethasone was used in 6 (60%) cases. One of them received Anakinra for 5 days and none of them received Tocilizumab. The comparison of the treatment and outcome between patients who died and survivors is detailed in Table 2.

## 3. Discussion

Recent studies have described the incidence, clinical and radiologic features, laboratory characteristics, and hyperinflammatory parameters, therapeutic strategies, recovery, evolution, outcome, and screening of COVID-19 in MHD patients [10, 11, 14–18, 23]. However, there are few studies on the technique and the dialyzer used in patients with COVID-19 undergoing hemodialysis [10, 19, 20], as well as on the response of this infection on the removal of the inflammatory parameters. We present a single-center experience of the use of the ATA membrane dialyzer in postdilution OL-HDF in 10 MHD patients with COVID-19. We used this dialyzer since it may reduce the concentration of inflammatory cytokines. In addition, it minimizes the loss of albumin, thus not altering the coagulation patterns. Furthermore, it may avoid adverse hypersensitivity reactions, reduce additional inflammation, and achieve a good clearance of uremic toxins. ATA membranes are characterized by presence of low electrical charge, allowing a low adsorption capacity [19], which influences the properties of antithrombotic agents described for these membranes [20].

The incidence of COVID-19 in our HD unit during the second pandemic wave was 13.2%, which is very similar compared to other cohorts [10, 14, 21, 23]. Mortality and complication rates are high in MHD patients. This is probably due to a very high number of comorbid conditions. We observed that 4 of the 10 patients presented a severe form of COVID-19. In our cohort, we found a 30% death rate in COVID-19-infected MHD patients, which is in line with other reports in Europe (29% in Italy and 28–31% in Spain), although higher than 16% reported in China [10, 14, 21, 22].

MHD patients with COVID-19 could be detected early because they are admitted to the centers of HD three times a week. This has allowed various biomarkers of inflammation to be proposed to predict severe forms of COVID-19 and mortality [24]. Elevation of common parameters associated with inflammation, such as CRP, procalcitonin (PCT), and cytokines, is very frequently found in COVID-19 [25, 26]. However, the NLR, PLR, MLR, and LCR are biomarkers of systemic inflammation, available in almost all laboratories, and are rarely used in this infection. The first of them has been the most thoroughly studied and has shown prognostic ability in multiple clinical settings, such as acute kidney injury [27], kidney disease [28], cardiovascular disease [29], ANCA-associated vasculitis [30], cancer patients [31], renal transplantation [32], and recently COVID-19 as a short-term predictor marker associated with severe forms of COVID-19 in HD patients [15, 18, 33]. In our study, the CRP levels at diagnosis were almost double in patients who died, as compared to survivors. Luo et al.[34] found in a study of 298 patients that CRP performed better than other parameters (age, neutrophil count, and platelet count) in predicting adverse outcomes. Admission serum CRP levels were identified as a moderate discriminator of disease severity. However, PCT levels in our patients at diagnosis were higher in survivors, as compared to deceased.

Several reports of non-MHD patients with COVID-19 have described elevated levels of neutrophils and CRP with lymphopenia [13, 23, 26], but very few reports have considered the cost-effective markers NLR, PLR, MLR, and LCR to aid complication predictions and response to the therapeutic measures performed. We observed that the blood test based on the combination of these biomarkers performed on Days 7 and 14, rather than at diagnosis (Day 0), better discriminates patients who will develop severe forms of COVID-19 and died, from those with the nonsevere forms and survivors. Out of all these ratios, in our study, NLR and LCR were associated with the severe form and mortality. Very similar observer data by Qin et al. [26] showed that an NLR >5.2 was associated with the most severe form of COVID-19 within a cohort of 452 patients, while Mutinelli-Szymanski et al. [15], in a prospective, observational, and multicentric study of 62 adult HD patients, observed that an NLR >3.7 at day 7 was associated with the severe form and with poor survival. Therefore, we believe that NLR and LCR could be helpful for clinicians to identify patients who may develop a severe form of the disease at an early stage.

Severe forms of COVID-19 induce an inflammatory reaction triggered by a cytokine storm, causing direct tissue damage and organ failure [25, 35]. Some studies have suggested that the dynamic change of IL-6 and IL-1 levels and other cytokines can be used as a marker in disease monitoring in patients with severe COVID-19 [36]. Del Valle et al.[37] investigated, in a study of 1814 nonhemodialysis patients of the Mount Sinai Health System in New York, several cytokines upon admission. The authors propose that serum IL-6 and tumor necrosis factor-alpha (TNF-*α*) levels should be considered in the management and treatment of patients with COVID-19 to stratify prospective clinical trials, guide resource allocation, and inform therapeutic options. In a recent Spanish observational, single-center, prospective study, Quiroga et al.[38] evaluated the impact of reducing IL-6 using a cytokine adsorbent filter in 16 COVID-19 hemodialysis patients. IL-6 levels were obtained before and after the first admission hemodialysis session and at 1 week. The authors concluded that a positive IL-6 balance during the admission hemodialysis session was associated with higher mortality. We observed in our study that at admission, predialysis IL-6 levels were very similar in patients who died, as compared to those who survived. However, on day 14, IL-6 levels were higher in patients who died. Despite having been prescribed an ATA dialyzer, patients who died did not show a decrease in IL-6 levels during the first 2 weeks after the diagnosis of COVID-19 compared to those who survived. However, we observed a 68.6% reduction in IL-6 levels in the surviving patients.

COVID-19 is associated with a syndrome of hypercoagulability with implications in the coagulation of extracorporeal hemodialysis circuits [39]. Nephrologists should consider using dialyzer membranes with poor platelet and coagulation activity in order to minimize the use of LMWH during the dialysis session and to maximize its efficacy with systemic anticoagulation for the management of the hypercoagulation state of this infection. Several studies have described the benefits of the use of LMWH in patients with COVID-19 for the prevention of activation of the coagulation cascade, induced by inflammation [40]. In addition, Perna et al.[41] recommend the use of LMWH in each hemodialysis session and propose to monitor these patients with Anti-Factor Xa activity assay and D-dimer levels to possibly increase the LMWH dosage. In our study, we monitor patients only with D-dimer levels. Patients during their follow-up presented a bad clinical respiratory course, associated with a progressive increase in these levels. In these patients, the dose of LMWH was increased. This underscores the need for a dialyzer membrane that requires little anticoagulation such as the ATA membrane, therefore maximizing the effectiveness of the LMWH.

Several studies have evaluated the coagulation parameter D-dimer in the progression of COVID-19. Bilaloglu et al.[42] observed, in 3334 consecutive patients admitted to 4 hospitals in New York City, that a thrombotic event occurred in 16.0%. Higher D-dimer levels at presentation were independently associated with thrombotic events, consistent with early coagulopathy. In our study, during COVID-19 infection, our MHD patients at diagnosis presented an increase in D-dimer of >1400 ng/mL. However, this increase did not correlate with mortality. Furthermore, in MHD hemodialysis patients infected with COVID-19, the D-dimer levels, even though they may not be higher than those of the non-COVID hemodialysis population, should be evaluated with caution due to the wide dispersion of values.

So far, only glucocorticoids have shown a beneficial effect in severe COVID-19 situations in controlled clinical trials [43, 44]. The usefulness of Tocilizumab in COVID-19 is being controversial, since its use in most retrospective studies has been associated with improved survival, but the result of different clinical trials does not seem favorable [45–47]. On the other hand, the use of inhibitory molecules of other interleukins is currently being studied, with Anakinra as the most commonly investigated anti-interleukin molecule studied after Tocilizumab for the treatment of COVID-19 [48]. In our case series, only one patient received treatment with Tocilizumab and, given the poor evolution, Anakinra was added. Despite having received both immunomodulatory treatments, the patient was one of the 3 patients who died. However, it is worth mentioning that the 3 patients who did not survive were patients with high comorbidity due to tumor history, autoimmune, and cardiovascular disease.

The main limitations of our study were the small sample size, the observational retrospective design, a possible selection bias, and the difficulty to assess the response in the decrease in inflammatory parameters with the use of ATA dialyzer, since at the moment there is no standard treatment regimen for this type of patients. Secondly, we collected IL-6 levels at the time of diagnosis and after 2 weeks. Therefore, we were only able to assess the removal of IL-6 in those patients who did not present a severe form of the disease. However, one of the strengths of our study is the measurement of the markers NLR, PLR, MLR, and LCR, which were carried out systematically every 7 days in all patients.

In conclusion, our understanding of treatment in COVID-19 has changed substantially in the past months, in both the nonrenal and renal population in HD. Some dialyzers, such as ATA membranes, may be helpful in the modulation of an immune dysregulated response in COVID-19 infection in MHD patients. Further observations and studies will be needed to fully understand the removal of inflammatory parameters with the use of these dialysis membranes in patients with COVID-19.

## Figures and Tables

**Table 1:** Patients characteristics, dialysis parameters, and clinical findings at COVID-19 diagnosis.

Characteristics	All patients (*n* = 10)	Death patients (*n* = 3)	Survivors (*n* = 7)	*p* value^*∗*^
*Demographics*				
Age (years)	71.5 [69.0–80.0]	69 [58–82]	72 [70–80]	0.45
Male, *n* (%)	5 (50)	2 (67)	3 (43)	0.50
Diabetes mellitus, *n* (%)	4 (40)	1 (33.3)	3 (42.9)	0.8
Hypertension, *n* (%)	10 (100)	3 (100)	7 (100)	1
Initial nephropathy, *n* (%)				0.35
Diabetic nephropathy	2 (20)	1 (33.3)	1 (14.3)	—
Hypertension	3 (30)	—	3 (42.9)	—
Chronic interstitial nephritis	3 (30)	1 (33.3)	2 (28.6)	—
Vasculitis	1 (10)	1 (33.3)	—	—
Amyloidosis	1 (10)	—	1 (14.3)	—
*Dialysis details*				
*Vascular access*, *n* (%)				
Tunneled CVCs	6 (60)	2 (66.7)	4 (57.1)	0.79
AVF native	3 (30)	1 (33.3)	2 (28.6)	—
Prosthetic fistula	1 (10)	—	1 (14.3)	—
ESA (UI/week)	12000 [9000–18000]	15000 [9000–15000]	9000 [6000–30000]	0.91
Dialysis time (h)	3.75 [3.5–4.0]	4.0 [3.5–4.0]	3.75 [3.5–4.0]	0.46
Dry weight (kg)	67.3 [0.54–73.5]	68.5 [49–70]	66.0 [54.0–76.0]	0.73
Ultrafiltration volume (ml)	1867 (1587–2145)	1494 [1422–1587]	1984 [1819–2235]	0.02
Blood flow rate (ml/min)	343.9 [322.5–350.3]	344.1 [322.5–346.6]	343.6 [321.6–350.4]	0.91
Dialysis vintage (month)	49.4 [30.7–62.7]	30.7 [12.3–56.2]	56.2 [37.1–91.9]	0.29
*Signs and symptoms*, *n* (%)				
Fever	6 (60)	3 (100)	3 (42.9)	0.09
Cough	7 (70)	3 (100)	4 (57.1)	0.18
Diarrhea	2 (20)	2 (66.7)	0	0.02
Bilateral pneumonia	4 (40)	3 (100)	1 (14.3)	0.04

Data are shown as median [interquartile range] or percentage (%). AVF: arteriovenous fistula; CVC: central venous catheter; ESA: erythropoiesis-stimulating agents. ^∗^Mann–Whitney *U* or Chi-square.

**Table 2:** Patients' treatment and outcome.

	All patients (*n* = 10)	Death patients (*n* = 3)	Survivors (*n* = 7)	*p* value^*∗*^
Antibiotic therapy, *n* (%)	6 (60)	3 (100)	3 (43)	0.2
Cephalosporin (third generation)	3 (30)	2 (50)	1 (14.3)	0.2
Macrolide	1 (10)	—	1 (14.3)	1
Carbapenems	4 (40)	3 (100)	1 (14.3)	0.03
Others	3 (30)	2 (66.7)	1 (14.3)	0.2
Steroids, *n* (%)	6 (60)	3 (100)	3 (43)	0.2
Anakinra, *n* (%)	1 (10)	1 (33.3)	0	0.3
Tocilizumab, *n* (%)	0	0	0	
High flow oxygen therapy >30 lpm, *n* (%)	4 (40)	3 (100)	1 (14.3)	0.03
Anticoagulation, *n* (%)	5 (50)	3 (100)	2 (28.6)	0.2

Data are shown as number and percentage. ^∗^Chi-square/Fisher's exact test.

**Table 3:** Inflammatory parameters at baseline, 7 days, and 14 days after diagnosis.

	At baseline	At 7 days	At 14 days	*p* value^*∗*^
CRP (mg/dL)	1.54 [0.52–4.31]	1.63 [0.49–12.89]	3.23 [0.35–8.49]	0.28
PCT (mg/dL)	0.54 [0.37–0.75]	0.88 [0.21–1.17]	0.42 [0.20–0.84]	0.54
Ferritin (ng/mL)	1020 [601–1952]	1639 [702–2958]	1706 [603.5–3286]	0.28
WBC count (10^3^/*μ*L)	4.8 [4.5–6.9]	7.4 [4.3–11.1]	8.7 [6.6–12.4]	0.007
NLR	3.1 [2.6–7.5]	5.8 [2.5–22]	13.3 [3.2–22.6]	0.04
LCR	1.7 [0.4–1.9]	0.65 [0.08–1.01]	0.9 [0.01–4.9]	0.88
MLR	2.3 [1.7–3.5]	2.5 [1.7–4.0]	2.7 [1.7–3.3]	0.33
Fibrinogen (mgl/dL)	529.5 [328–610]	530 [411–718]	491.5 [468–517]	0.58
IL-6 (pg/mL)	36.0 [28.4–70.3]		80.6 [11.3–220.4]	0.721

^∗^Wilcoxon matched-pairs signed-ranks test (14 days vs baseline). CRP: C-reactive protein; PCT: procalcitonin; WBC: white blood cell; NLR: neutrophil-to-lymphocyte ratio; LCR: lymphocyte-to-C-reactive protein ratio; MLR: monocyte-lymphocyte ratio; IL-6: interleukin 6.

**Table 4:** Laboratory data at diagnosis according to vital status.

	All patients (*n* = 10)	Death patients (*n* = 3)	Survivors (*n* = 7)	*p* value^*∗*^
Hemoglobin (g/dL)	10.7 [10.5–11.9]	10.6 [8.3–11.9]	10.8 [10.5–13.8]	0.49
ALT (UI/L)	12.5 [10.0–15.0]	13.0 [10.0–170]	12.0 [10.0–15.0]	0.56
AST (UI/L)	18 [14–22]	16 [14–17, 23]	28 [23–2064]	0.02
NT-proBNP (pg/dL)	3926 [3200–9145]	9145 [2500–10023]	3900 [3200–4760]	0.57
Troponin (g/l)	60 [48.7–80.9]	80.9 [56.1–81.0]]	60.0 [44–60.7]	0.3
GGT (UI/L)	23.0 [15.0–38.0]	30.0 [17.0–38.0]	18.0 [14.0–38.0]	0.49
D-dimer (ng/dL)	1489 [780–2476]	1753 [426–1982]	1225 [780–3302]	0.73
CPK (UI/L)	59 [31–81]	81.0 [23.0–780]	58 [31–80]	0.43
Fibrinogen (mgl/dl)	529 [328–610]	531 [275–651]	528 [328–610]	0.91
IL-6 (pg/ml)	36.0 [28.4–70.3]	41.4 [31.8–95.4]	31.1 [21.3–70.3]	0.31
WBC (10^3^/*μ*L)	4.8 [4.5–6.9]	5.4 [4.5–9.5]	4.8 [4.3–6.9]	0.31
MLR	2.3 [1.7–3.5]	2.5 [1.4–55.3]	2.0 [1.7–3.5]	0.91
NLR	3.1 [2.6–7.5]	2.7 [2.6–6.5]	3.3 [2.5–9.4]	0.57
LCR	1.7 [0.4–1.9]	0.4 [0.3–0.6]	1.8 [1.6–2.1]	0.09
Ferritin (ng/mL)	1020 [601–1952]	1062 [978–19083]	915 [336–1952]	0.31
PCT (mg/dL)	0.54 [0.37–0.75]	0.54 [0.33–100]	0.53 [0.37–0.75]	0.91
CRP (mg/dL)	1.5 [0.5–4.3]	1.7 [1.7–7.9]	0.8 [0.3–4.3]	0.21

^∗^Mann–Whitney *U*. ALT: alanine aminotransferase; AST: aspartate aminotransferase; NT-proBNP: NT-proB-type natriuretic peptide; GGT: gamma-glutamyl transferase; CPK: creatine phosphokinase; IL-6: interleukin 6; WBC: white blood cell; NLR: neutrophil-to-lymphocyte ratio; LCR: lymphocyte-to-C-reactive protein ratio; MLR: monocyte-lymphocyte ratio; PCT: procalcitonin; CRP: C-reactive protein.

## Data Availability

The data used to support the findings of this study are available from the corresponding author on request.
